# Revolutionizing Cardiac Care: A Comprehensive Narrative Review of Cardiac Rehabilitation and the Evolution of Cardiovascular Medicine

**DOI:** 10.7759/cureus.46469

**Published:** 2023-10-04

**Authors:** Atta Ullah, Mahendra Kumar, Mohammad Sayyar, FNU Sapna, Chris John, Siraj Memon, Kashifa Qureshi, Elsie C Agbo, Henry I Ariri, Emmanuel J Chukwu, Giustino Varrassi, Mahima Khatri, Satesh Kumar, Naji M. Elder, Tamam Mohamad

**Affiliations:** 1 Internal Medicine, Cavan General Hospital, Cavan, IRL; 2 Internal Medicine, Khyber Teaching Hospital, Peshawar, PAK; 3 Medicine, Sardar Patel Medical College, Bikaner, IND; 4 Medicine, Khyber Medical College, Peshawar, PAK; 5 Pathology, Albert Einstein College of Medicine, Bronx , USA; 6 Internal Medicine, University College Dublin, Dublin, IRL; 7 Medicine, Liaquat University of Medical & Health Sciences, Jamshoro, PAK; 8 Internal Medicine, Kyiv Medical University, Kyiv, UKR; 9 Internal Medicine, All Saints University School of Medicine, Roseau, DMA; 10 Pain Medicine, Paolo Procacci Foundation, Rome, ITA; 11 Medicine and Surgery, Dow University of Health Sciences, Karachi, PAK; 12 Medicine and Surgery, Shaheed Mohtarma Benazir Bhutto Medical College, Karachi, PAK; 13 Medicine, Santa Clara University, Santa Clara, USA; 14 Cardiovascular Medicine, Wayne State University, Detroit, USA

**Keywords:** evolution in cardiac treatment, rehabilitation programs, cardiovascular disease, revolutionizing cardiac care, cardiovascular medicine, cardiac rehabilitation

## Abstract

Cardiovascular disease (CVD) stands as a global health crisis, with its complex web of conditions, including coronary artery disease, heart failure, hypertension, and stroke, continuing to exact a heavy toll on individuals and healthcare systems worldwide. Despite substantial advances in medical technology and pharmaceutical interventions, CVD remains a formidable adversary, necessitating innovative prevention, management, and rehabilitation approaches. In tracing the historical trajectory of CVD, the narrative reveals the antiquated practices of early 20th-century medicine, marked by extended bed rest as the primary modality for heart-related conditions. It underscores the critical juncture when exercise was first recognized as a therapeutic tool for cardiac health, setting the stage for the evolution of cardiac rehabilitation (CR). CR programs have transcended their initial focus on exercise, expanding to encompass dietary guidance, psychosocial support, and comprehensive risk factor modification. These holistic interventions enhance physical recovery and address the psychosocial and lifestyle aspects of CVD management, ultimately improving patients' overall well-being. CR programs increasingly leverage advanced technologies and personalized strategies to tailor interventions to individual patient needs, ultimately enhancing outcomes and reducing the burden of CVD. In conclusion, this narrative review illuminates the transformative journey of cardiac care, with a particular spotlight on the indispensable role of CR in reshaping the landscape of cardiovascular medicine. By evolving from historical practices to comprehensive, patient-centered interventions, CR has made significant strides in improving the prognosis, quality of life, and holistic well-being of individuals grappling with the complexities of CVD. Understanding this historical context and the contemporary advancements is paramount for healthcare professionals and policymakers as they navigate the intricate terrain of cardiovascular medicine and endeavor to mitigate the impact of this pervasive disease.

## Introduction and background

Cardiovascular disease (CVD) is a formidable global health challenge, demanding our unwavering attention and innovative solutions. As we stand on the precipice of the third decade of the 21st century, CVD is the leading cause of morbidity and mortality worldwide. The World Health Organization (WHO) estimated that 17.9 million lives were prematurely claimed by CVD in 2016, accounting for 31% of all global deaths [1]. This staggering statistic underscores the urgency of our collective efforts to address the multifaceted challenges CVD poses. CVD is an umbrella term encompassing a spectrum of conditions, including coronary artery disease, heart failure, hypertension, stroke, and peripheral artery disease. These conditions share a common thread of affecting the heart or blood vessels, with devastating consequences for individuals, families, and healthcare systems. The impact of CVD extends beyond clinical medicine, reaching into public health, economics, and social well-being. CVD affects individuals across the lifespan, from congenital heart conditions in infants to atherosclerotic diseases in the elderly. It presents an intricate web of risk factors, including genetic predisposition, lifestyle choices, and environmental influences. The modifiable risk factors, such as poor diet, physical inactivity, smoking, and excessive alcohol consumption, have led to an epidemiological transition where CVD is no longer confined to affluent nations but has become a global epidemic [2].

The economic burden of CVD is equally alarming. A report by the American Heart Association (AHA) estimated that the direct and indirect costs of CVD and stroke in the United States reached a staggering $351.2 billion in 2014 [3]. This financial burden places immense strain on healthcare systems, public resources, and individual households, underscoring the urgent need for effective prevention and management strategies. Furthermore, CVD is not merely a statistical abstraction. It is a deeply personal and often life-altering experience for those who live with it. Patients with CVD often face a trajectory marked by hospitalizations, invasive procedures, chronic medication regimens, and physical limitations. The psychological toll of CVD, including anxiety, depression, and reduced quality of life, cannot be overstated [4]. Moreover, the burden of CVD disproportionately affects vulnerable populations, including racial and ethnic minorities, individuals with low socioeconomic status, and those living in underserved regions [5]. These disparities in CVD outcomes highlight the urgency of addressing both clinical and social determinants of health. Cardiovascular medicine has witnessed a remarkable evolution in the face of this formidable challenge. While medical advancements have led to improved diagnostic tools, medications, and interventional procedures, one facet of CVD care has emerged as a transformative force - cardiac rehabilitation (CR). CR is rooted in a fundamental shift in our understanding of CVD. Historically, the prevailing approach to managing heart disease was characterized by extended bed rest and limited therapeutic options. However, the paradigm shifted when pioneers recognized the potential of exercise as a therapeutic modality for heart health. The American College of Cardiology (ACC) defines CR as "a comprehensive exercise, education, and behavior modification program designed to improve the physical and emotional condition of patients with heart disease" [6]. CR is a structured, evidence-based intervention encompassing supervised exercise, dietary counseling, risk factor modification, and psychosocial support. Over the decades, CR programs have evolved from their nascent beginnings to become a cornerstone of cardiovascular care. They have expanded their scope beyond exercise alone, adopting a holistic approach that addresses the multifaceted dimensions of CVD. CR programs now emphasize lifestyle modifications, stress management, and patient education, recognizing that optimal cardiac care extends beyond prescription medications and surgical interventions [7].

In recent years, CR programs have embraced the principles of personalized medicine and precision cardiology. Advanced technologies, such as wearable devices and telehealth platforms, have been integrated into CR to tailor interventions to individual patient needs. This transformation aligns with the broader shift in healthcare toward patient-centered care and evidence-based practice [8]. The narrative review presented here embarks on a captivating journey through the annals of cardiovascular medicine. Its primary focus is to illuminate the transformative role of CR in the evolution of cardiac care. We will traverse the historical landscape of CVD management, starting from the era of bed rest and limited therapeutic options, and follow the trajectory of CR's emergence as a pivotal force in modern cardiac care. This narrative review will delve into the historical origins of CR, tracing its roots to early 20th-century practices. We will explore key milestones and developments that have shaped the field of CR, from the initial recognition of exercise as a therapeutic tool to the comprehensive, patient-centered programs today. Additionally, this review will highlight the shift from disease-centered care to holistic, patient-centered approaches in cardiovascular medicine. We will emphasize the importance of addressing physical health and psychosocial and lifestyle factors in CVD management. Moreover, we will discuss the integration of advanced technologies and personalized strategies in CR programs, reflecting the broader paradigm shift toward precision medicine in healthcare. By the end of this narrative review, readers will gain a comprehensive understanding of how CR has revolutionized cardiac care and contributed significantly to improving the prognosis, quality of life, and overall well-being of individuals affected by CVD. This knowledge is crucial for healthcare professionals, policymakers, and researchers as they navigate the complex landscape of cardiovascular medicine and work toward mitigating the impact of this pervasive and relentless disease. In the subsequent sections, we will delve deeper into the historical progression of cardiac care, the evolution of CR programs, the holistic approach to cardiac care, precision medicine in CR, the impact of CR on cardiovascular medicine, and the challenges and future directions of this transformative field.

## Review

Methods

Search Strategy

A narrative and comprehensive search was conducted across multiple electronic databases, including PubMed, MEDLINE, Scopus, Web of Science, and Google Scholar. These databases encompass medical literature, including peer-reviewed articles, reviews, and historical documents. A combination of medical subject headings (MeSH terms) and keywords was employed to optimize the search strategy. Relevant terms included "cardiac rehabilitation," "cardiovascular medicine," "cardiovascular disease," "history of medicine," "evolution of cardiac care," and related synonyms.

Inclusion Criteria

Studies and articles were considered for inclusion if they contributed to the historical context, development, or evolution of CR and its impact on cardiovascular medicine. While the primary focus was on peer-reviewed research, historical documents, books, and authoritative reports were included to provide a comprehensive narrative. No publication date limits were imposed during the search process to ensure a thorough exploration of the historical evolution of cardiac care.

Data Collection

The search results were initially screened based on titles and abstracts to identify articles and documents that were relevant to the topic. Studies that did not align with the scope of the narrative review were excluded at this stage. Following the initial screening, selected articles and documents underwent a full-text review. This rigorous assessment aimed to ensure that the included sources provided valuable insights into the historical progression of cardiac care and the role of CR.

Data Synthesis

This review follows a narrative approach, allowing for qualitatively synthesizing historical and contemporary evidence. Information extracted from the selected sources was organized chronologically and thematically to construct a coherent narrative of the evolution of CR and its impact on cardiovascular medicine. Special attention was given to contextualizing the historical developments within their respective times' broader socio-economic, cultural, and medical landscapes. Key themes, milestones, and innovations in cardiac care and rehabilitation evolution were identified and discussed in detail. These themes included the recognition of exercise as a therapeutic tool, the development of structured CR programs, the expansion of CR beyond exercise, psychosocial support, dietary counseling, risk factor modification in contemporary CR, and the integration of precision medicine approaches.

Assessment Criteria

Given the narrative nature of this review, formal quality assessment tools, such as those commonly used for systematic reviews, were not applied. Instead, sources were evaluated based on relevance, historical accuracy, and credibility. Historical information and interpretations were cross-referenced to ensure accuracy and reliability with authoritative historical accounts, expert opinions, and consensus statements in cardiovascular medicine.

Historical perspective of CVD

CVD has a deep-rooted history that spans millennia, evolving from a relatively unknown condition to the leading global cause of morbidity and mortality. This section delves into the historical context of CVD, its prevalence, and its profound impact on public health. We will also explore the early approaches to CVD management, shedding light on the limitations and challenges patients and healthcare professionals face.

Ancient Observations and Early Notions

The history of cardiovascular disease can be traced back to ancient civilizations, where vague references to heart ailments are found in ancient Egyptian and Greek medical texts. Hippocrates, often regarded as the father of medicine, made observations about chest pain and palpitations, though the understanding of these symptoms remained rudimentary [8]. The ancient Egyptians believed the heart to be the seat of intelligence and emotion. At the same time, the Greeks saw it as a complex organ responsible for circulating vital pneuma (breath) throughout the body. These early notions laid the foundation for later understanding but needed more precision and depth of modern cardiology [8].

The Renaissance and Advances in Cardiology

During the Renaissance, anatomists such as Andreas Vesalius made significant strides in understanding the human cardiovascular system. Vesalius's detailed anatomical drawings of the heart and circulatory system marked a turning point in cardiac science, allowing for a more accurate depiction of the heart's structure. In the 17th century, they witnessed the development of rudimentary instruments like the stethoscope and the recognition of auscultation as a diagnostic tool. These innovations were followed by the formulation of blood circulation theories by William Harvey in the early 17th century, which revolutionized the understanding of how blood moves through the heart and vessels [9].

Industrialization and the Rise of Cardiovascular Disease

The Industrial Revolution of the 18th and 19th centuries brought about profound changes in lifestyle, diet, and exposure to environmental risk factors, contributing to a surge in CVD. Urbanization, sedentary work, and dietary shifts towards high-calorie, high-sugar diets became prevalent, laying the groundwork for an epidemic of heart disease. The late 19th century saw the emergence of conditions like angina pectoris and myocardial infarction as recognized clinical entities. Physicians began to recognize the symptoms of chest pain, shortness of breath, and irregular heartbeats as indicative of heart disease, although precise diagnoses remained elusive [10].

Early Treatment Approaches and Limitations

Before the 20th century, therapeutic interventions for heart disease were limited and often ineffective. Bed rest was the standard recommendation for patients with heart conditions, with little understanding of the benefits of physical activity. Medications were scarce and needed more scientific validation, leaving patients with few options for treatment. Surgical interventions for heart disease were virtually nonexistent in the pre-20th century era. The first successful cardiac surgery, a pericardiectomy, was performed by Ludwig Rehn in 1896 [9]. However, cardiac surgery remained experimental and high-risk for many years. In summary, the historical perspective of CVD reflects a journey from ancient observations and rudimentary notions to significant advancements during the Renaissance and the recognition of CVD as a public health crisis during the Industrial Revolution. Early approaches to CVD management were limited by a need for more understanding, effective treatments, and surgical interventions, underscoring the need for further developments in cardiac care.

Emergence of CR

CR has a history that spans centuries, evolving from humble beginnings to becoming a cornerstone of modern cardiovascular care. This essay traces the origins of CR, detailing when and how exercise was first recognized as a therapeutic tool for heart health. It also describes the initial developments in structured CR programs and their early impact on patient outcomes. This historical journey sheds light on the foundations of contemporary CR. CVD has plagued humanity throughout history, but it was not until recently that organized efforts to rehabilitate individuals with heart conditions emerged. The roots of CR can be traced back to the early 20th century, although rudimentary forms of exercise therapy for heart patients existed before that. The emergence of CR as a distinct medical discipline marked a transformative moment in managing cardiovascular diseases [11].

Early Recognition of Exercise for Heart Health

The notion that exercise could benefit heart health has ancient roots. In various cultures, physical activity was linked to overall well-being, and some historical references hint at its role in promoting cardiovascular health. The ancient Greeks recognized the importance of physical activity for maintaining health. Hippocrates, often regarded as the father of modern medicine, promoted exercise as part of a healthy lifestyle [12]. Traditional Chinese practices like Tai Chi and Qigong emphasize body and mind harmonizing through slow, controlled movements and deep breathing. While not explicitly targeting heart health, these practices contributed to overall fitness. Medical observations of the therapeutic potential of exercise for heart conditions began to emerge in the 18th and 19th centuries. In the 19th century, physicians like Caleb Hillier Parry noted that physical exertion could affect the heart rate and rhythm. However, these observations were relatively isolated and lacked systematic application. Sir James Mackenzie, a Scottish cardiologist, made pioneering contributions to cardiovascular medicine. He developed innovative instruments to record heart sounds and electrical activity. Mackenzie's work contributed to the understanding of arrhythmias and the role of exercise testing in diagnosing heart conditions [13].

Early Developments in Structured CR Programs

Structured CR programs as we know them today began to take shape in the early 20th century, primarily driven by the work of dedicated healthcare professionals who recognized the potential benefits of exercise-based interventions. Dr. Arthur Stanley, a physician at the Royal Infirmary in Dundee, Scotland, is often credited with pioneering early CR efforts in the 1920s. Stanley developed a systematic approach to exercise-based rehabilitation for heart patients. His program involved graded exercise and a regimen that progressively increased patients' activity levels [14]. The devastating impact of myocardial infarction (heart attack) on patients' lives became a driving force for developing structured CR programs. Dr. Paul Dudley White, a renowned American cardiologist, advocated for post-MI rehabilitation. He recognized the potential for exercise therapy to aid recovery and improve patients' functional capacity. Dr Jeremy Morris, a British epidemiologist, conducted influential research demonstrating exercise's benefits in reducing the risk of coronary heart disease. His studies provided crucial evidence supporting the integration of exercise into CR. The early CR programs showed promising, relatively modest results compared to contemporary standards. Patients participating in structured CR programs reported improved exercise tolerance and functional capacity. They could perform daily activities with less fatigue and discomfort [15]. Early CR programs also recognized the psychosocial aspects of recovery. Patients often experienced improved mental well-being and increased confidence in managing their condition. The emergence of CR can be traced through historical references, early medical observations, and the pioneering work of healthcare professionals. Exercise-based rehabilitation efforts in the early 20th century, notably those driven by figures like Dr. Arthur Stanley, laid the foundation for today's comprehensive CR programs [14]. While the impact on patient outcomes was modest compared to modern CR, these early developments represented a crucial step in managing cardiovascular diseases. They highlighted the potential benefits of exercise therapy for heart health and set the stage for further advancements in CR.

Evolution of CR programs

CR has evolved remarkably over the decades, progressing from exercise-focused programs to comprehensive, multidimensional interventions. This essay examines the transformation of CR programs over time, including their expansion beyond exercise to encompass dietary counseling, psychosocial support, and risk factor management. We will discuss critical milestones and innovations that have shaped the modern CR landscape, highlighting the significance of these developments in improving cardiovascular care. CR programs have evolved significantly since their inception in the early 20th century [13]. Initially, CR primarily consisted of exercise-based interventions for individuals recovering from cardiovascular events. However, recognizing the multifaceted nature of cardiovascular health, CR has expanded to encompass a comprehensive approach addressing dietary, psychosocial, and risk factor management aspects.

Transformation of CR Programs

The earliest CR programs, such as those pioneered by Dr. Arthur Stanley in the 1920s, primarily emphasized graded exercise regimens. Patients engaged in supervised physical activities aimed at improving cardiovascular fitness and stamina. As the understanding of cardiovascular diseases deepened, it became evident that addressing risk factors and the psychological aspects of recovery was essential. This shift from exercise-centric to multidimensional care marked a pivotal moment in the evolution of CR programs [16]. Including dietary counseling in CR programs recognizes the crucial role of nutrition in cardiovascular health. Dietitians and nutritionists guide heart-healthy eating habits, addressing issues like cholesterol management, salt intake, and weight control. Psychosocial support has become an integral part of CR. Patients recovering from cardiovascular events often experience anxiety, depression, and stress. Psychologists and counselors assist individuals in coping with emotional challenges and improving their overall mental well-being. CR programs have adopted a proactive approach to managing risk factors. This includes interventions related to smoking cessation, blood pressure control, diabetes management, and medication adherence. The goal is to reduce the modifiable risk factors contributing to cardiovascular disease [17].

Key Milestones and Innovations

The Framingham Heart Study, initiated in 1948, was pivotal in identifying cardiovascular risk factors. It led to a paradigm shift in CR programs, emphasizing the importance of addressing these risk factors. The study's findings highlighted the significance of high blood pressure, elevated cholesterol levels, and smoking as crucial contributors to heart disease [18]. Establishing guidelines by organizations like the American Heart Association (AHA) and the American Association of Cardiovascular and Pulmonary Rehabilitation (AACVPR) provided a framework for CR programs. These guidelines helped standardize care, ensuring patients received evidence-based interventions tailored to their needs [19]. Advancements in technology have revolutionized CR. Wearable devices and telehealth platforms enable remote monitoring of patients' vital signs and exercise regimens. These innovations enhance accessibility and adherence to CR programs, particularly in remote or underserved areas. The concept of personalized medicine has gained prominence in CR. Risk stratification tools help identify patients at higher risk of future cardiovascular events. Personalized exercise prescriptions, medication regimens, and lifestyle recommendations are tailored to individual patient profiles [20]. Psychosocial support has evolved by integrating cognitive-behavioral therapy, stress management, and mindfulness techniques into CR programs. These interventions address mental health and the mind-body connection in cardiovascular recovery [17]. The evolution of CR programs from exercise-centric regimens to comprehensive, multidimensional interventions reflects an understanding of the complex nature of cardiovascular health. Dietary counseling, psychosocial support, and risk factor management have become integral components, significantly improving patient outcomes and quality of life. Key milestones and innovations, such as the Framingham Heart Study, guideline development, technological advancements, personalized medicine, and psychosocial interventions, have collectively shaped the modern CR landscape. These advancements continue to enhance cardiovascular care and reduce cardiovascular disease burden on individuals and society.

Holistic approach to cardiac care

There has been a profound transformation in cardiac care in recent years, shifting from a traditional disease-centered model to a holistic, patient-centered approach. This essay delves into the holistic approach to cardiac care, emphasizing the importance of addressing physical health and psychosocial and lifestyle factors in managing CVD. We will explore the reasons behind this paradigm shift, the components of a holistic approach, and its impact on patient outcomes. Historically, the management of CVD primarily focused on treating the physical manifestations of the disease, such as reducing cholesterol levels or controlling blood pressure. However, this approach often neglected the broader aspects of patient well-being, including psychosocial and lifestyle factors. Recognizing the limitations of the disease-centered model, healthcare professionals have increasingly adopted a holistic approach to cardiac care [21].

Reasons for the Paradigm Shift

CVD is a complex, multifactorial condition influenced by genetic, environmental, and behavioral factors. A disease-centered approach needs to address the intricate variables contributing to CVD risk and progression. The shift towards holistic care aligns with the principles of patient-centered care. It recognizes patients as unique individuals with diverse needs, preferences, and values. Holistic care places the patient at the center of decision-making, ensuring care plans are tailored to their circumstances [20]. Research has highlighted the substantial impact of psychosocial and lifestyle factors on cardiovascular health. Stress, depression, social support, physical activity, and dietary choices significantly influence CVD outcomes. Ignoring these elements can hinder the effectiveness of traditional treatments [21].

Components of a Holistic Approach

Holistic cardiac care places a strong emphasis on addressing psychosocial factors. Psychologists, counselors, and support groups play crucial roles in helping patients cope with the emotional and psychological aspects of living with CVD. Managing stress, anxiety, and depression can improve well-being and enhance treatment plan adherence. Lifestyle factors are pivotal in CVD management. Holistic care incorporates lifestyle modification as a cornerstone of treatment. This includes dietary counseling to promote heart-healthy eating patterns, smoking cessation programs, physical activity recommendations, and weight management strategies. Patients actively set realistic goals and make sustainable lifestyle changes [17]. Shared decision-making between healthcare providers and patients is fundamental in a holistic approach. Patients are encouraged to participate in their care actively, making informed decisions about their treatment options. This collaborative approach fosters a sense of ownership and empowerment in managing their condition. Holistic care involves a comprehensive assessment of a patient's risk factors, not limited to traditional cardiovascular markers. This may include evaluating sleep patterns, social determinants of health, and overall quality of life. A holistic risk assessment provides a more complete picture of a patient's health status [22].

Impact on Patient Outcomes

Holistic care enhances patient engagement and adherence to treatment plans. When patients are actively involved in setting goals and addressing psychosocial and lifestyle factors, they are more likely to comply with medical recommendations, resulting in better outcomes. Addressing psychosocial and lifestyle factors contributes to an improved quality of life for individuals with CVD. Patients experience reduced stress, enhanced emotional well-being, and greater satisfaction with their overall health [20]. Studies have demonstrated that holistic approaches to cardiac care can lead to reduced rates of cardiovascular events. Lifestyle modifications, psychosocial support, and shared decision-making collectively improve long-term outcomes [23]. The shift from a disease-centered model to a holistic, patient-centered approach in cardiac care marks a significant advancement in managing cardiovascular disease. Recognizing the complexity of CVD and the profound impact of psychosocial and lifestyle factors, healthcare professionals have embraced a more comprehensive approach to care. This approach, which integrates psychosocial support, lifestyle modification, shared decision-making, and thorough risk assessment, improves patient outcomes and enhances the overall quality of life for individuals living with CVD. Holistic cardiac care represents a paradigm shift prioritizing patients' well-being and individual needs, ultimately leading to more effective and compassionate cardiovascular management.

Precision medicine in CR

In recent years, precision medicine has emerged as a transformative approach to healthcare. This essay delves into integrating advanced technologies and personalized strategies in CR programs. We will explore how precision medicine is applied to tailor interventions to individual patient needs and optimize outcomes, focusing on cardiovascular health. CVD is a complex and heterogeneous group of conditions requiring personalized treatment and rehabilitation approaches [3]. Precision medicine, often called customized therapy, recognizes individuals' unique genetic, lifestyle, and environmental factors influencing their health. In CR, precision medicine seeks to maximize the effectiveness of interventions by customizing them to each patient's specific needs [24].

Integration of Advanced Technologies

Precision medicine in CR begins with a comprehensive analysis of a patient's genetic and genomic profile. Advances in genomics have allowed for the identification of genetic variants associated with CVD risk. Genetic testing can identify individuals with a heightened genetic predisposition to certain cardiovascular conditions, enabling targeted interventions and risk reduction strategies. Wearable devices, such as smartwatches and fitness trackers, have become integral to precision medicine in CR. These devices provide real-time data on a patient's heart rate, physical activity, sleep patterns, and more. Remote monitoring allows healthcare providers to track patient progress and adjust treatment plans accordingly [25]. AI and machine learning algorithms analyze large datasets to identify patterns and trends in CVD risk and outcomes. These technologies assist in risk stratification, predicting individual patient responses to treatment, and optimizing rehabilitation plans. AI-driven decision support systems help healthcare providers make data-informed choices. Pharmacogenomics considers an individual's genetic makeup when prescribing medications [26]. This approach helps identify the most effective and safest medicines for a particular patient. In CR, pharmacogenomic analysis can optimize medication regimens for hypertension or dyslipidemia.

Personalized Strategies in CR Programs

Precision medicine in CR involves designing exercise programs tailored to an individual's physical capacity, preferences, and goals. Genetic information can influence exercise recommendations, such as aerobic or resistance training, intensity, and duration. Dietary recommendations are personalized based on genetic and metabolic factors. Nutrigenomics assesses how an individual's genes influence their nutritional needs. Precision nutrition plans address dietary choices, portion sizes, and nutrient intake to optimize cardiovascular health [15]. Psychosocial factors play a significant role in CVD recovery. Precision medicine considers an individual's psychological profile and stressors when designing psychosocial interventions. Counseling and support programs are tailored to address specific emotional and mental health needs [16]. Pharmacogenomic insights guide medication selection and dosing. Precision medicine ensures patients receive the most effective medications, reducing adverse effects and optimizing therapeutic outcomes [26].

Optimizing Outcomes

Precision medicine optimizes the efficacy of CR interventions. Tailored exercise, nutrition, and medication regimens are more likely to lead to positive outcomes, including improved cardiovascular fitness, better medication adherence, and enhanced overall well-being. Advanced technologies and genetic insights enable more accurate risk stratification. Patients at higher risk of recurrent cardiovascular events can receive intensified interventions, while those at lower risk may benefit from less intensive programs, reducing the overall burden on healthcare resources [27]. Data collected from wearable devices and genetic testing enable data-driven decision-making by healthcare providers. Real-time monitoring allows for timely adjustments to treatment plans, ensuring that patients receive the most appropriate and effective care [27].

Ethical and Practical Considerations

Integrating advanced technologies in CR raises critical ethical considerations regarding patient data privacy and security. Strict safeguards must be in place to protect sensitive genetic and health information. Ensuring equitable access to precision medicine in CR is crucial. Disparities in access to genetic testing and advanced technologies must be addressed to prevent healthcare inequalities [28]. Precision medicine represents a paradigm shift in CR, promising highly personalized and effective interventions. Integrating advanced technologies, genetic insights, and customized strategies empowers healthcare providers to optimize outcomes for individuals with cardiovascular disease. By tailoring exercise, nutrition, psychosocial support, and medication management to each patient's unique needs, precision medicine is revolutionizing CR and ushering in a new era of personalized cardiovascular care.

Impact of CR on cardiovascular medicine

CR has emerged as a cornerstone of cardiovascular medicine, offering a multifaceted approach to improving the lives of individuals with CVD. This essay explores the substantial impact of CR on CVD prognosis, quality of life, and overall well-being. It also delves into the role of CR in reducing the burden of CVD on healthcare systems and society. CVD remains a leading cause of mortality worldwide. However, cardiovascular medicine has witnessed significant advancements in managing CVD, with CR emerging as a pivotal component of comprehensive care. CR programs encompass exercise training, risk factor management, dietary counseling, psychosocial support, and education to optimize cardiovascular health [4,5].

Positive Impact of CR on CVD Prognosis

CR leads to substantial improvements in functional capacity, as measured by exercise tolerance and fitness levels. Patients experience enhanced exercise capacity, allowing them to perform daily activities with reduced effort and fatigue. CR programs comprehensively address modifiable cardiovascular risk factors, such as hypertension, dyslipidemia, and diabetes. CR contributes to risk reduction and improved cardiovascular health through risk factor management and education. A study published in the Journal of the American College of Cardiology (2016) reported that participation in CR significantly reduced systolic and diastolic blood pressure, promoting better blood pressure control [29].

Enhancing Quality of Life and Overall Well-Being

CVD often takes a toll on the psychosocial well-being of individuals. Depression, anxiety, and stress are common among CVD patients. CR programs integrate psychosocial support and counseling to address these issues, leading to improved mental health. CR interventions extend beyond physical health, emphasizing improving overall quality of life. Patients report improved social functioning, reduced symptom burden, and greater confidence in managing their condition. CR programs empower individuals with the knowledge and skills to make sustainable lifestyle changes. This includes guidance on heart-healthy eating habits, smoking cessation, and increased physical activity [27].

Reducing the Burden on Healthcare Systems and Society

CR programs offer substantial economic benefits by reducing hospital readmissions and the overall cost of care. Preventing recurrent cardiovascular events through CR saves lives and reduces the financial burden on healthcare systems. The positive effects of CR extend to society as a whole. By improving the health and well-being of individuals with CVD, CR contributes to a healthier and more productive population. CR helps individuals regain their independence and reduces the need for long-term disability support, ultimately benefiting society by maintaining an effective workforce [30]. CR has emerged as a transformative force in cardiovascular medicine, significantly impacting CVD prognosis, quality of life, and overall well-being. Evidence and data consistently support the role of CR in reducing the risk of cardiovascular events, improving functional capacity, enhancing psychosocial well-being, and promoting healthy lifestyles. Moreover, CR programs offer economic benefits by reducing healthcare costs and contributing to a healthier and more productive society. As a cornerstone of comprehensive cardiovascular care, CR continues revolutionizing the field, offering hope and improved outcomes to individuals with CVD and society.

Challenges and future directions

CR has demonstrated its effectiveness in improving the lives of individuals with CVD. However, there are still challenges and barriers to its widespread adoption and implementation. This essay acknowledges these challenges and provides insights into future developments in CR and their implications for cardiovascular medicine. CVD remains a global health challenge, necessitating innovative approaches to prevention and management. CR has emerged as a cornerstone of cardiovascular care, offering multifaceted interventions to improve the health and well-being of CVD patients. However, several challenges hinder its broader adoption, and future directions are essential to further enhance its impact on cardiovascular medicine.

Challenges in CR Adoption and Implementation

One of the primary challenges in CR is the need for more utilization of available programs. Despite its proven benefits, many eligible patients do not participate in CR, often due to a lack of awareness, transportation issues, or logistical barriers. A study found that only 20-30% of eligible patients participated in CR programs after a cardiac event, highlighting the underutilization issue [26]. Access to CR programs is not uniform across regions, leading to geographic disparities in care. Rural and underserved areas often lack CR facilities, limiting access for those most need it. The financial aspects of CR present challenges for both patients and healthcare systems. Inadequate insurance coverage and reimbursement policies can hinder program sustainability and patient participation [27]. Psychosocial factors, such as depression, anxiety, and low motivation, can deter individuals from engaging in CR. Identifying and addressing these barriers is crucial for successful rehabilitation. Comprehensive CR programs that integrate psychosocial support have been shown to mitigate these barriers [17].

Future Directions in CR

Telehealth and remote monitoring technologies have gained prominence, especially in light of the COVID-19 pandemic. Future CR programs may increasingly utilize telehealth platforms to provide remote consultations, monitor patient progress, and deliver exercise regimens. The integration of precision medicine into CR will further enhance its effectiveness. Genetic and genomic data and advanced risk stratification tools will enable tailored interventions that address individual patient needs. As discussed in a previous section, precision medicine is poised to revolutionize CR by customizing exercise regimens, dietary plans, and medication management based on genetic and clinical profiles [28]. Home-based CR programs offer flexibility and convenience for patients. Future developments may expand home-based options, allowing patients to participate in CR without frequent visits to healthcare facilities. Behavioral interventions, including cognitive-behavioral therapy, motivational interviewing, and digital health apps, will be more prominent in CR. These interventions address psychosocial factors and enhance patient engagement [29].

Implications for Cardiovascular Medicine

Future developments in CR will improve patient outcomes, including reduced cardiovascular events, enhanced quality of life, and better management of CVD risk factors. As CR becomes more personalized and accessible, its impact on cardiovascular medicine will continue to grow. Wider adoption of CR and cost-effective approaches like telehealth and home-based programs will contribute to cost savings for healthcare systems. Preventing costly cardiovascular events through CR is a cost-effective strategy. Future directions in CR should prioritize addressing geographic and socioeconomic disparities in access. Telehealth and home-based programs can help bridge gaps in care, ensuring that all individuals have equal access to CR. CR represents a critical component of cardiovascular medicine, offering a holistic approach to improving the health and well-being of individuals with CVD. While challenges in adoption and implementation persist, future developments in CR promise to transform cardiovascular care. Telehealth, precision medicine, home-based programs, and behavioral interventions will shape the future of CR, offering more personalized, accessible, and effective interventions. As CR continues to evolve, it will play an increasingly pivotal role in reducing the burden of cardiovascular disease on individuals, healthcare systems, and society.

Limitations

Acknowledging the limitations inherent in this narrative review is imperative to evaluate the findings appropriately. Selection bias in including papers and studies is duly acknowledged as a primary concern. Despite diligent attempts to have a comprehensive array of sources, there remains a potential for unintentionally overlooking pertinent studies or advancements. Moreover, it is essential to acknowledge the potential presence of publication bias in this review, given the tendency for studies reporting favorable outcomes to be more readily published than those with negative or equivocal results. Additionally, it is crucial to acknowledge the temporal extent of this review. The literature search was performed through September 2021, potentially excluding any advancements in CR and cardiovascular medicine subsequent to that period. The potential emergence of new research discoveries, technology breakthroughs, and clinical guidelines may have implications for the relevance and comprehensiveness of this review.

Furthermore, the issue of generalizability poses a significant barrier in the context of this review. Although our objective was to offer a thorough examination, it is essential to note that the results may not entirely capture the range of CR programs and methods found in different countries and healthcare settings. It is necessary to exercise caution when extrapolating these findings to encompass all patient demographics and healthcare environments. Furthermore, there is variation in the quality of the studies and the incorporated sources. Certain studies may have limitations concerning their study design, sample size, or methodological rigor. Therefore, the inferences derived from this analysis depend on the caliber of the foundational evidence. Moreover, the intrinsic problem of CR lies in its highly customized nature. The variability of outcomes and effectiveness is contingent upon patient characteristics, comorbidities, and program components. The review may only partially capture the entirety of this heterogeneity, underscoring the necessity for customized methods in clinical practice.

## Conclusions

In conclusion, this review underscores CR's vital role in reshaping cardiovascular medicine. After a thorough literature analysis, CR is shown to significantly enhance the quality of life for heart patients, leading to reduced mortality, improved cardiac function, and enhanced psychological well-being. Over time, CR has evolved, embracing innovative technologies, personalized exercise plans, and holistic lifestyle interventions, addressing various aspects of cardiovascular health. CR's importance cannot be overstated; it aids recovery and prevents future cardiac issues. Its enduring role in cardiovascular medicine emphasizes staying at the forefront of research and adapting to healthcare changes. CR's commitment to tailoring interventions to individual needs ensures its continued prominence. As we progress, CR will remain a cornerstone of cardiovascular care, contributing to superior outcomes, enriched patient experiences, and a brighter future for heart patients. Its holistic approach, encompassing physical, psychological, and social well-being, reaffirms its indispensable status in cardiac care.
